# Supplementary material for: Efficacy and safety of single-step transepithelial photorefractive keratectomy with the all-surface laser ablation SCHWIND platform without mitomycin-C for high myopia: A retrospective study of 69 eyes
Source: PLoS One. 2021 Dec 7;16(12):e0259993. doi: 10.1371/journal.pone.0259993 (PMC8651116; doi:10.1371/journal.pone.0259993)
Supplement: Attachment — Submitted filename: PONE-D-21-13895_reviewer 1.doc [file pone.0259993.s001.doc]

This is a meaning research about efficacy and safety of single-step transepithelial photorefractive keratectomy with the all surface laser ablation SCHWIND platform without mitomycin-C for high myopia. The major conclusion is suggested that ASLA-SCHWIND TransPRK without mitomycin-C appears to be safe as well as effective and accurate for high myopia. The research in general is meaningful. However, there are a number of issues that need to be attentioned.

In the part of case presentation:

1.The detail of how to define the corneal haze measurementse is not described in the paper. I think it will be better to add the Fantes Grades standard.

2.In the paper, all consecutive eyes with high myopia (≤-6 D) that were treated in 2018–2020 with the 39 SCHWIND Amaris 500E® TransPRK excimer laser without adjuvant mitomycin-C in a 40 tertiary-care hospital (France) and were followed up for 3 months were identified. Is the range of high myopia(≤-6 D) correct in the method right?

3.Since some patients appear haze at 6 month, so it is really short of 3 months follow-up. So there could be a risk of false negatives in the results.

Jinjin Zhang
