# Supplementary material for: Efficacy and safety of single-step transepithelial photorefractive keratectomy with the all-surface laser ablation SCHWIND platform without mitomycin-C for high myopia: A retrospective study of 69 eyes
Source: PLoS One. 2021 Dec 7;16(12):e0259993. doi: 10.1371/journal.pone.0259993 (PMC8651116; doi:10.1371/journal.pone.0259993)
Supplement: Attachment — Submitted filename: plos one response reviewers.docx [file pone.0259993.s002.docx]

**Response to Reviewers and Editors**

**Journal Requirements**

1. Please ensure that your manuscript meets PLOS ONE's style requirements, including those for file naming.

- We have reformatted the manuscript to meet PLOS One style requirements.

2. Please provide additional details regarding participant consent for use of participant's anonymized data for research purposes. In the ethics statement in the Methods and online submission information, please describe whether verbal consent was informed, how verbal consent was documented and witnessed, and why written consent was not obtained. If your study included minors, state whether you obtained consent from parents or guardians.

- We included the following text to the Ethics section: “The consent procedure was conducted in accordance with the reference methodology MR-004 of the National Commission for Information Technology and Liberties of France (No. 588909 v1).”

3. We note that you have stated that you will provide repository information for your data at acceptance. Should your manuscript be accepted for publication, we will hold it until you provide the relevant accession numbers or DOIs necessary to access your data. If you wish to make changes to your Data Availability statement, please describe these changes in your cover letter and we will update your Data Availability statement to reflect the information you provide

- The dataset has been deposited in Zenodo. The DOI is 10.5281/zenodo.5507429.

4. We note that you have included the phrase “data not shown” in your manuscript. Unfortunately, this does not meet our data sharing requirements. PLOS does not permit references to inaccessible data. We require that authors provide all relevant data within the paper, Supporting Information files, or in an acceptable, public repository. Please add a citation to support this phrase or upload the data that corresponds with these findings to a stable repository (such as Figshare or Dryad) and provide and URLs, DOIs, or accession numbers that may be used to access these data. Or, if the data are not a core part of the research being presented in your study, we ask that you remove the phrase that refers to these data.

- We have replaced the text “data not shown” with the visual acuity of the two patients who had to undergo surgical revision because they lost 2–3 lines of visual acuity after PRK: “The retreatment resulted in satisfactory outcomes (0.1 and 0 logMAR) and no corneal haze 6 months after revision surgery.”

**Additional Editor Comments**

The reviews have identified some significant flaws that need to be addressed prior to making decision about publication. One of the reviewers feel that study design is poor required attention specially when it comes to Post PRK Haze. A minimum 6 months or more follow-up of patients is required. This has been identified a major flaw. Authors need to include additional data or provide solid evidence that it is not necessary. Other concerns expressed by reviewers are given below.

- We have supplied the 6 month follow-up data for not only post-PRK haze but also other variables. As a result, Tables 2 and 3 and Figures 1–4 were revised along with the relevant text in the Abstract and Results section of the manuscript.

Post-PRK haze was also not observed at 6 months.

**Reviewers' comments**

Reviewer #1: This is a meaning research about efficacy and safety of single-step transepithelial photorefractive keratectomy with the all surface laser ablation SCHWIND platform without mitomycin-C for high myopia. The major conclusion is suggested that ASLA-SCHWIND TransPRK without mitomycin-C appears to be safe as well as effective and accurate for high myopia. The research in general is meaningful. However, there are a number of issues that need to be attentioned.

- Thank you very much for your thoughtful consideration of our paper. We have addressed your comments to the best of our ability.

In the part of case presentation:1.The detail of how to define the corneal haze measurementse is not described in the paper. I think it will be better to add the Fantes Grades standard.

- We added the Fantes Grade to Figure 3.

2.In the paper, all consecutive eyes with high myopia (≤-6 D) that were treated in 2018–2020 with the 39 SCHWIND Amaris 500E® TransPRK excimer laser without adjuvant mitomycin-C in a 40 tertiary-care hospital (France) and were followed up for 3 months were identified. Is the range of high myopia(≤-6 D) correct in the method right?

- Yes, all eyes treated are < or = to -6D.

3.Since some patients appear haze at 6 month, so it is really short of 3 months follow-up. So there could be a risk of false negatives in the results.

- We have supplied the 6 month follow-up data for not only post-PRK haze but also other variables. As a result, Tables 2 and 3 and Figures 1–4 were revised along with the relevant text in the Abstract and Results section of the manuscript.

Post-PRK haze was also not observed at 6 months.

Reviewer #2: I congratulate the Authors on this wonderful study.

- Thank you very much for your thoughtful consideration of our paper. We have addressed your comments to the best of our ability.

This is an important research question when it comes to Post PRK haze and Mitomycin- C.
However the Study design is poor and requires to be framed properly.
Also When it comes to Post PRK Haze, the minimum follow- up required is 6 months or above, which is lacking pre-dominantly in the manuscript

- We have supplied the 6 month follow-up data for not only post-PRK haze but also other variables. As a result, Tables 2 and 3 and Figures 1–4 were revised along with the relevant text in the Abstract and Results section of the manuscript.

Post-PRK haze was also not observed at 6 months.
